# Mechanistic study of PpIX accumulation using the JFCR39 cell panel revealed a role for dynamin 2-mediated exocytosis

**DOI:** 10.1038/s41598-019-44981-y

**Published:** 2019-06-17

**Authors:** Yuya Kitajima, Takuya Ishii, Takeo Kohda, Masahiro Ishizuka, Kanami Yamazaki, Yumiko Nishimura, Tohru Tanaka, Shingo Dan, Motowo Nakajima

**Affiliations:** 1 SBI Pharmaceuticals Co., Ltd, Tokyo, Japan; 2 Division of Molecular Pharmacology, Cancer Chemotherapy Center, Japan Foundation for Cancer Research, Tokyo, Japan

**Keywords:** Enzyme mechanisms, Cancer imaging

## Abstract

5-aminolevulinic acid (5-ALA) has recently been employed for photodynamic diagnosis (ALA-PDD) and photodynamic therapy (ALA-PDT) of various types of cancer because hyperproliferating tumor cells do not utilize oxidative phosphorylation and do not efficiently produce heme; instead, they accumulate protoporphyrin IX (PpIX), which is a precursor of heme that is activated by violet light irradiation that results in the production of red fluorescence and singlet oxygen. The efficiencies of ALA-PDD and ALA-PDT depend on the efficient cellular uptake of 5-ALA and the inefficient excretion of PpIX. We employed the JFCR39 cell panel to determine whether tumor cells originating from different tissues can produce and accumulate PpIX. We also investigated cellular factors/molecules involved in PpIX excretion by tumor cells with the JFCR39 cell panel. Unexpectedly, the expression levels of ABCG2, which has been considered to play a major role in PpIX extracellular transport, did not show a strong correlation with PpIX excretion levels in the JFCR39 cell panel, although an ABCG2 inhibitor significantly increased intracellular PpIX accumulation in several tumor cell lines. In contrast, the expression levels of dynamin 2, which is a cell membrane-associated molecule involved in exocytosis, were correlated with the PpIX excretion levels. Moreover, inhibitors of dynamin significantly suppressed PpIX excretion and increased the intracellular levels of PpIX. This is the first report demonstrating the causal relationship between dynamin 2 expression and PpIX excretion in tumor cells.

## Introduction

Surgery is a typical modality that is used for cancer therapy along with chemotherapy and radiotherapy and has the advantage of reliably resecting cancer tissue. While it is true that the precision of resection and the detection of small metastatic lesions are strongly related to patient prognosis^1–3^, it is often difficult to distinguish the boundary region between normal and cancerous tissues. A simple clinical method to discriminate between normal and cancer tissues has been eagerly anticipated.

Diagnostic imaging techniques for cancer, such as computed tomography (CT), magnetic resonance imaging (MRI), and positron emission tomography (PET), have been widely adopted, and their effectiveness has been confirmed. However, large expensive instruments are required for these imaging techniques, and real-time and *in situ* observations are difficult to make with these techniques; therefore, there is a limit to their usage. Real-time, *in situ* imaging technology based on optical principles such as narrow band imaging (NBI) or indocyanine green (ICG)-dependent imaging techniques have also been clinically applied. However, these techniques were developed for the detection of blood and lymphatic vessels but not for the detection of cancer cells^4,5^. Fluorescent reagents and similar detectable compounds that specifically accumulate in cancer cells are being developed as probes for visualizing cancer cells; however, most of those technologies have not yet been utilized in clinical practice^6,7^.

On the other hand, 5-aminolevulinic acid (5-ALA)-dependent photodynamic diagnosis (ALA-PDD), which is also a technology used for directly detecting cancer cells, has been utilized for some types of cancer, and clinical studies on many kinds of cancer have also been reported. Stummer *et al*.^8^ reported that progression-free survival (PFS) was prolonged when ALA-PDD was used during glioblastoma surgery, and 5-ALA has already been approved as a diagnostic drug for grade III and IV glioma in Europe, Japan and the USA^9,10^. Recently, 5-ALA was also approved as a diagnostic agent for nonmuscle invasive bladder cancer in Japan^11^. Furthermore, the usefulness of ALA-PDD has been reported for a wide range of cancers, such as the peritoneal metastasis of ovarian cancer^12^, gastric cancer^13^, colon cancer^14^, the peritoneal dissemination of gastric cancer^13^, hepatic cancer^15^, lung cancer^16^, breast cancer^17^, and cervical cancer^18^.

ALA-PDD and ALA-PDT^19^ are a cancer cell detection method and a cancer therapy technology, respectively, that use 5-ALA. 5-ALA is an amino acid commonly synthesized in mitochondria in cells, in which it is used as the sole source for heme biosynthesis. When applied externally for the purpose of ALA-PDD or ALA-PDT, 5-ALA is taken up by active transporters in the cell membrane, such as PEPT1, PEPT2, GAT2, TAUT, and PAT1^20–22^, and metabolized by heme synthesis enzymes, and 8 molecules of 5-ALA are converted to protoporphyrin IX (PpIX). Finally, ferrochelatase (FECH) incorporates Fe^2+^ into PpIX to generate heme. However, in cancer cells, heme biosynthesis is incomplete due to the unique metabolic features of cancer cells. In contrast to normal differentiated cells, which primarily rely on mitochondrial oxidative phosphorylation to generate the energy needed for cellular processes, most cancer cells instead rely on aerobic glycolysis, which consumes a large amount of glucose and produces excess lactate, resulting in acidosis. This phenomenon is termed “the Warburg effect” and causes a lack of NADH and the insufficient conversion of Fe^3+^ to Fe^2+^, resulting in low FECH activity and the consequent accumulation of PpIX, especially when an abundant amount of 5-ALA is exogenously administered^23^. PpIX is a fluorescent substance that emits red fluorescence at a peak wavelength of 635 nm when excited with violet light near 400 nm, corresponding to the Soret band. ALA-PDD is a real-time bioimaging technology that makes it possible to visualize only living cancer cells on the basis of this property. On the other hand, PpIX is activated by red light and causes phototoxicity, which is utilized for ALA-PDT in tumor cells. There are still many factors to be considered in the detailed mechanisms involved in the accumulation of PpIX by cancer cells, and thus extensive research is currently being carried out. At the level of basic research, many reports have been published on the accumulation of PpIX in different types of human cancer cell lines derived from a wide variety of tissues, such as brain tumor^24^, hepatic cancer^25^, gastric cancer^26^, melanoma^27^, ovarian cancer^28^, cervical cancer^29^, breast cancer^30^, prostate cancer^31^, colon cancer^32^, lung cancer^33^, bladder cancer^34^, oral cancer^35^, pancreatic cancer^36^, esophageal cancer^37^ and osteosarcoma^38^.

The accumulation of PpIX in cancer cells is related to the balance between the generation of PpIX from 5-ALA in cells and the excretion of PpIX outside cells. Hagiya *et al*.^39^ reported that the balance between the protein expression level of PEPT1, which is a peptide transporter that is involved in the uptake of ALA into cells, and the expression level of ATP binding cassette subfamily G member 2 (ABCG2), which is a drug-resistant transporter involved in the elimination of PpIX, was correlated with the levels of PpIX accumulation in several gastric cancer cell lines. They also showed that ABCG2 inhibitors suppress the extracellular excretion of PpIX and increase its intracellular accumulation.

There have been many publications on various cancer cell lines that have demonstrated the intracellular accumulation of PpIX. However, there have been no reports of the comprehensive and simultaneous evaluation of the amount of PpIX accumulation in various types of cancer cells under identical conditions. The JFCR39 cell panel is a panel of 39 human cancer cell lines from 9 different tissues established by the Japanese Foundation for Cancer Research that is based on the NCI60 cell panel developed by the NCI, NIH, USA. The JFCR39 cell panel was used along with an anti-cancer drug sensitivity database and a gene expression database. Therefore, it is useful for not only disease-oriented screening but also the prediction of the modes of action and molecular targets of a given antitumor agent. For example, there have been several reports on the efficacy and characterization of PI3K inhibitors and on the search for targeted signaling pathways based on the JFCR39 cell panel data^40–42^.

Here, we measured the amount of PpIX accumulation after the administration of ALA in each cell line in the JFCR39 cell panel. In addition, we investigated the involvement of the PpIX efflux mechanism as an important factor for PpIX accumulation and evaluated cell susceptibility to excretion transporter inhibitors.

## Materials and Methods

### Chemicals and reagents

5-aminolevulinic acid (5-ALA) was obtained from Neo ALA (Tokyo, Japan). Fumitremorgin C (FTC) and an antibiotic antimycotic solution were purchased from Sigma (St. Louis, USA). RPMI 1640 and dimethyl sulfoxide were purchased from Wako (Osaka, Japan). FBS was purchased from Biowest (Nuaillé, France). The dynamin inhibitor toolbox, myristyl trimethyl ammonium bromide (MiTMAB™), ABCG2 antibody (Mouse monoclonal [BXP-21] to BCRP/ABCG2, ab3380), and dynamin 2 antibody were purchased from Abcam (Cambridge, UK). Alexa 680 goat anti-rabbit IgG (H + L) and Alexa 680 goat anti-mouse IgG (H + L) were purchased from Life Technologies (Carlsbad, USA).

### Cancer cell lines

A panel of 39 human cancer cell lines (JFCR39 cell panel) that was described previously^43–45^ was used for the *in vitro* experiments. Cells were cultured in RPMI 1640 medium supplemented with 5% fetal bovine serum (FBS) and incubated at 37 °C in 5% CO_2_.

### Fluorescence microscopy

The cells were seeded in a 35-mm dish and incubated with 1 mM 5-ALA at 37 °C for 4 h. Then, the medium was replaced with PBS (-). Images of PpIX fluorescence in cells in the culture dish were obtained using a CKN41 fluorescence microscope (Olympus, Tokyo, Japan) equipped with a DP73 digital camera.

### Measurement of intracellular and extracellular PpIX accumulation in JFCR39 cell panels

Cells from the JFCR39 cell panel were seeded in a 96-well plate with a black wall and a clear bottom and cultured at 37 °C for 24 h. After 24 h of culture, the cancer cells were incubated with 10 to 1,000 μM 5-ALA with or without 10 μM FTC in RPMI 1640 with 5% FBS at 37 °C for 4 h. Since the serum-dependent export of protoporphyrin IX by ATP-binding cassette transporter G2 (ABCG2) has been reported^46^, the experiments were carried out in the presence of 5% FBS. To measure extracellular PpIX accumulation, the cell culture supernatants were transferred to another 96-well plate with black walls and a clear bottom. To measure intracellular PpIX accumulation, the cells were washed twice with PBS and lysed in 100 μL of 1% SDS solution. The PpIX accumulation was determined on the basis of the fluorescence intensity using a EnVision 2103 microplate reader (PerkinElmer, Waltham, USA) (excitation wavelength: 405 nm, emission wavelength: 635 nm).

### Protein assay

The protein contents in the PpIX measurement samples were determined using a BCA Protein Assay Kit (Thermo Fisher Scientific, Tokyo, Japan). The protein assay was performed according to the standard procedure used for microplates. Then, the absorbance at 562 nm was measured using an Infinite M200PRO microplate reader (Tecan Japan, Kawasaki, Japan).

### Preparation of the total cell extract

The preparation of the total cell extract for Western blot analysis was performed as described previously^40^. Briefly, cells were resuspended in lysis buffer composed of 10 mM Tris-HCl, pH 7.4, 50 mM NaCl, 0.5% w/v NP40, 0.1% w/v SDS, 50 mM sodium fluoride, 30 mM sodium pyrophosphate, 50 mM sodium orthovanadate, 5 mM EDTA, 0.1 unit/mL trypsin inhibitor aprotinin, and 1 mM phenylmethylsulfonyl fluoride and lysed by sonication in an ice bath. The concentrations of the proteins in the extracts were determined using a BCA Protein Assay Kit.

### Western blot analysis

The Western blotting analysis was performed as described previously^40^. The samples were prepared by using 10 μg of total protein and subjected to 5–10% SDS-PAGE. Then, the separated proteins were transferred onto an Immobilon FL polyvinylidene difluoride membrane (Merck Millipore, Burlington, USA). The membrane was incubated in Odyssey® Blocking Buffer (PBS) (Merck Millipore, Burlington, USA) for 1 h at room temperature to block nonspecific binding and probed overnight with primary antibodies with the following dilutions: 1:1000 for the ABCG2 antibody and 1:2000 for the dynamin 2 antibody. After overnight incubation with primary antibody at 4 °C, the membrane was washed three times with 0.1% Tween 20-PBS (PBST) and treated with Alexa Fluor 680 anti-mouse IgG or anti-rabbit IgG antibody for 1 h at room temperature. Thereafter, the membrane was washed three times with PBST. The specific protein signals from the bound labeled antibodies were visualized with an Odyssey Infrared Imaging System (LI-COR Biosciences, Lincoln, USA).

### PpIX accumulation in cancer cells after incubation with 5-ALA and a dynamin inhibitor

Cells were seeded in a 96-well plate with black walls and a clear bottom and cultured in 5% CO_2_ at 37 °C for 24 h. Then, the cancer cells were incubated with 1 mM 5-ALA or 1 mM 5-ALA and 1 μM to 16 μM MiTMAB in RPMI1640 supplemented with 5% FBS at 37 °C in the dark for 4 h. PpIX extraction was performed as described in the abovementioned method. The fluorescence intensity of PpIX was measured using a Tecan Infinite M200PRO microplate reader (Tecan Japan, Kawasaki, Japan) (excitation wavelength: 405 nm, emission wavelength: 635 nm).

### PpIX accumulation in JFCR39 cells after incubation with ALA with or without FTC and dynamin inhibitor

Cells were seeded in a 96-well plate and cultured at 37 °C for 24 h. Then, the JFCR39 cells were incubated with 1 mM 5-ALA, 10 μM FTC and 8 μM MiTMAB in RPMI1640 supplemented with 5% FBS at 37 °C in the dark for 4 h. PpIX extraction was performed as described in the methods. The fluorescence intensity of PpIX was measured using an EnVision 2103 microplate reader (PerkinElmer) (excitation wavelength: 405 nm, emission wavelength: 635 nm).

## Results

### Intracellular and extracellular accumulation of PpIX after the administration of 5-ALA in the JFCR39 cell panel

**Figure 1:**
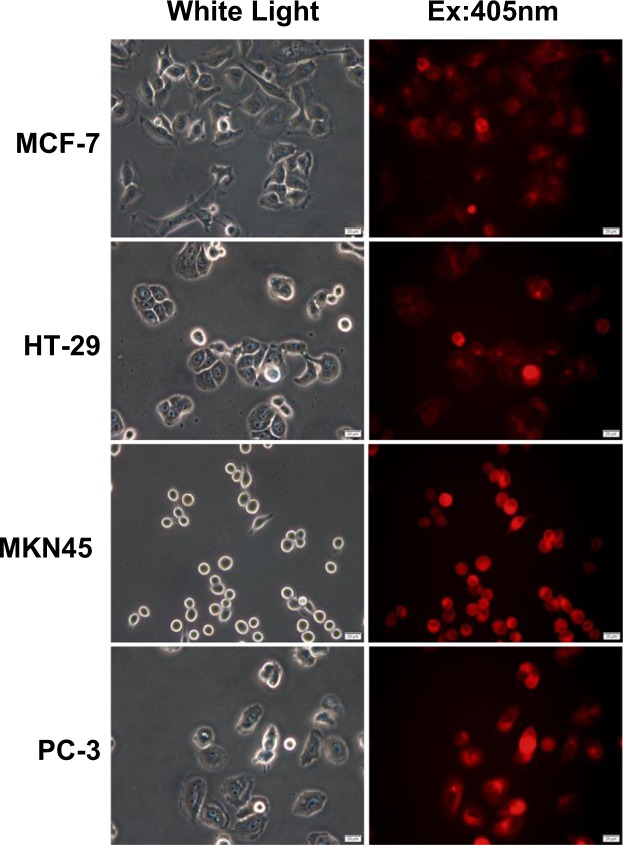
Accumulation of PpIX after administration of 5-ALA in cancer cells Cancer cells from cell lines MCF-7, HT-29, MKN45 and PC-3 were incubated with 1 mM 5-ALA for 4 h. PpIX fluorescence was detected by fluorescence microscopy. Magnification is 400X.

We first confirmed the possibility of 5-ALA-derived PpIX accumulation in representative cancer cell lines. Four human cancer cell lines selected from the JFCR39 panel were incubated with 1 mM 5-ALA. After 4 h of incubation, we could clearly detect red fluorescence from PpIX in cancer cells by fluorescence microscopy (Fig. 1) but not in human normal cells, such as umbilical vein endothelial cells, as has been previously reported (data not shown)^47^. In both MCF-7 and HT-29 cells, only a portion of the cells exhibited fluorescence. Rounded, non-attached cells exhibited greater fluorescence than adhered, flat cells.

**Figure 2:**
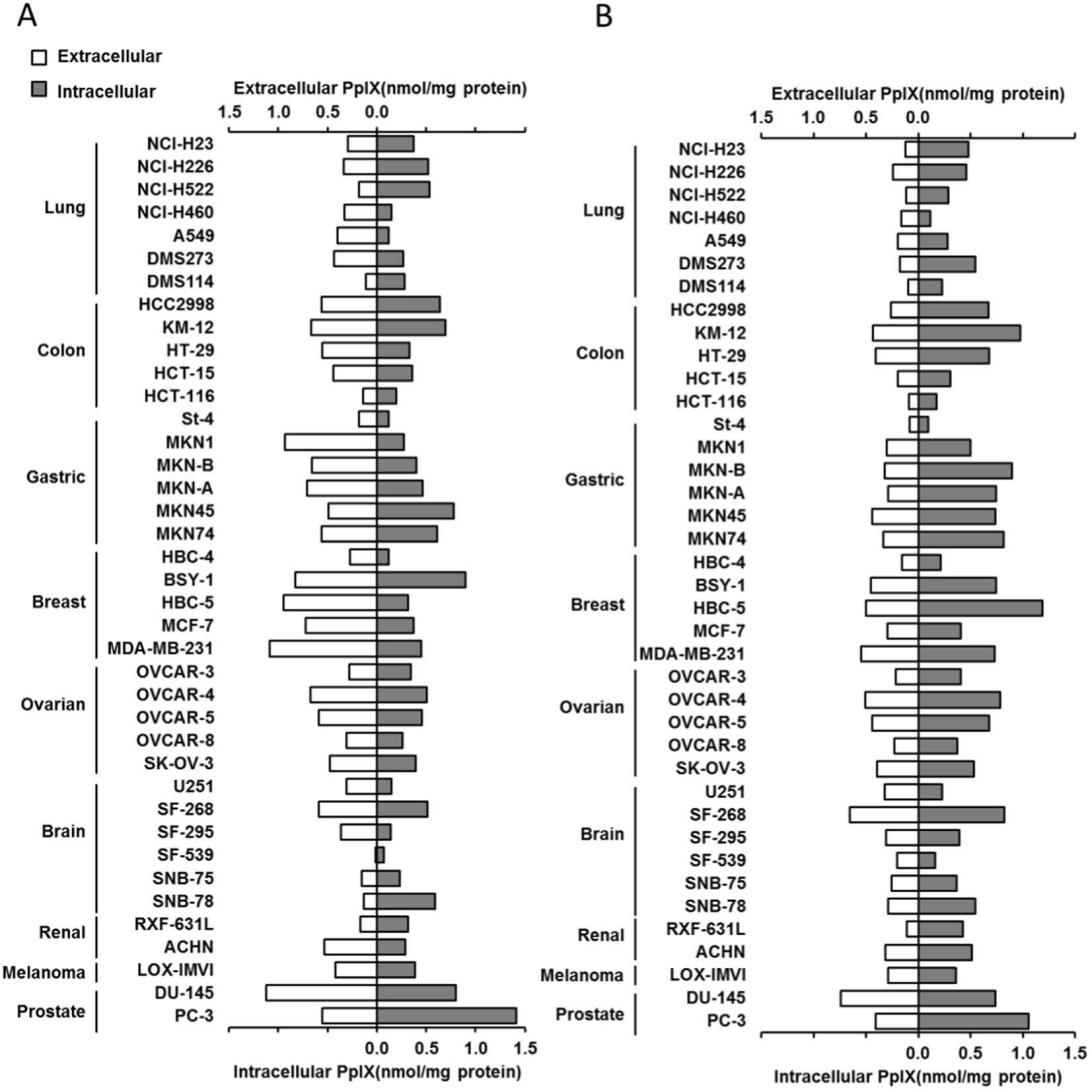
PpIX accumulation in cells from the JFCR39 cell panel after treatment with 5-ALA or 5-ALA and FTC (**A**) Intracellular and extracellular PpIX accumulation after incubation with 1 mM 5-ALA. (**B**) Intracellular and extracellular PpIX accumulation after incubation with 1 mM 5-ALA and 10 μM FTC.

Then, we performed the assay in all 39 cell lines in the JFCR39 cell panel. The JFCR39 cells were incubated with 10 μM to 1 mM 5-ALA. All cell lines in the JFCR39 cell panel showed the accumulation of PpIX in cells in a dose-dependent manner (data not shown). PpIX was also detected in the culture supernatants from all cell lines, demonstrating that the cells had specific mechanisms that allowed them to excrete PpIX across the cell membrane. Figure 2A shows the intracellular and extracellular amounts of PpIX in each cell line when the cells were incubated with 1 mM 5-ALA for 4 h.

We next investigated the effects of transporter inhibitors on the distribution of PpIX. JFCR39 cells were incubated with or without 10 μM fumitremorgin C (FTC), which is an inhibitor of the ATP-dependent transporter ABCG2, as shown in Fig. 2A,B, respectively. FTC treatment did not significantly change the total PpIX amounts in general; however, in almost all cells, the intracellular PpIX levels increased, while the extracellular PpIX levels decreased, as shown in Fig. 2A,B. These results were consistent with those in previous reports that demonstrated that ABCG2 is the main pathway involved in the excretion of PpIX. Although FTC generally reduced PpIX excretion, the sensitivity of cells to FTC differed widely among the cell lines (Fig. 2A,B), suggesting the involvement of other mechanisms in PpIX excretion in addition to those underlying ABCG2-mediated transport.

### Western blot analysis of ABCG2 and dynamin 2

**Figure 3:**
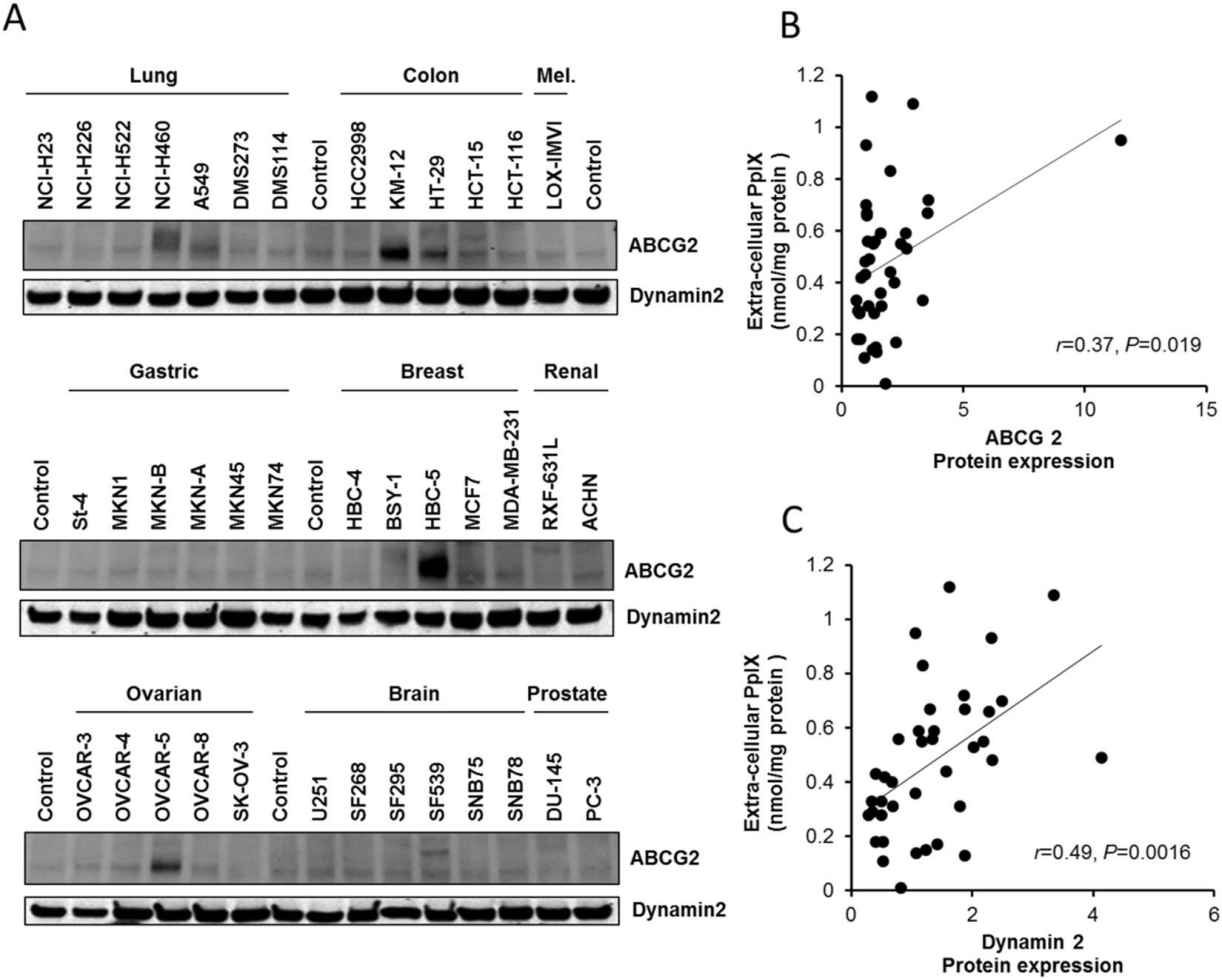
Western blot analysis of ABCG2 and dynamin 2 (**A**) ABCG2 and dynamin 2 protein expression in the JFCR39 panel cell lines. (**B**) Correlation between ABCG2 protein expression and the extracellular PpIX level. (**C**) Correlation between dynamin 2 protein expression and the extracellular PpIX level. (P < 0.005). The Pearson correlation coefficients (r) and the P values (P) are shown.

PpIX excretion by ATP-dependent transporters has been well investigated, and there have been many publications that have suggested that ABCG2 is most likely the transporter that regulates PpIX excretion. To investigate the correlation between the cellular capacity to excrete PpIX and ABCG2 expression levels, we examined ABCG2 protein expression in JFCR39 cells by Western blot analysis. ABCG2 expression levels varied widely among the cell lines (Fig. 3A); however, there was a correlation between ABCG2 expression and PpIX efflux (r = 0.37, P = 0.019) (Fig. 3B).

Since the expression level of ABCG2 did not appear by itself to fully explain the mechanism involved in PpIX excretion, we hypothesized that PpIX excretion could be mediated by mechanisms other than those involving ABC transporters, such as exocytosis. To examine this hypothesis, we investigated the expression of dynamin 2 in JFCR39 cells and the relationship between its expression levels and extracellular PpIX levels in the cell lines in the JFCR39 cell panel. Dynamin 2 expression was detected in all 39 cell lines, and its expression levels were significantly correlated with extracellular PpIX levels (Fig. 3A,C) (r = 0.49, P = 0.0016). These results suggested the involvement of the exocytosis pathway in PpIX excretion.

### Effects of dynamin inhibitors on PpIX accumulation

**Figure 4:**
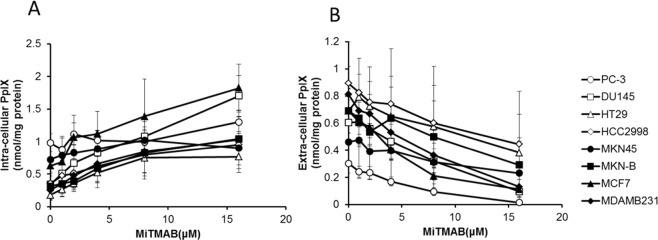
Effects of dynamin inhibitors on PpIX accumulation Cancer cells were incubated with 1 mM 5-ALA for 4 h in the presence of 1 μM to 16 μM MiTMAB, and then intracellular and extracellular PpIX accumulation was determined. (**A**) Intracellular PpIX; (**B**) extracellular PpIX. Data are expressed as the means ± S.D. of multiple experimental replicates (n = 3).

Since the results of the protein expression analysis strongly suggested that exocytosis was a pathway that was possibly involved in PpIX excretion, we attempted to investigate the hypothesis directly by using exocytosis inhibitors. Myristyl trimethyl ammonium bromide (MiTMAB) is an inhibitor of dynamin 2 that inhibits the GTPase activity of dynamin 2 by interrupting the interaction between the PH domain of the dynamin 2 protein and phospholipids in the cell membrane. It has been reported that MiTMAB inhibits dynamin-dependent exocytosis^48^. We selected 8 cell lines (2 cell lines each from colon cancer, gastric cancer, breast cancer, and prostate cancer) and incubated these with 1 mM 5-ALA and various doses of MiTMAB. Then, the intracellular and extracellular PpIX contents were measured (Fig. 4). In the 8 tested cell lines, MiTMAB treatment increased intracellular PpIX levels and simultaneously decreased extracellular PpIX levels in a dose-dependent manner. These findings strongly supported our hypothesis that dynamin-dependent exocytosis could be an important pathway involved in the excretion of PpIX from cells.

We also examined other dynamin inhibitors to determine whether they could inhibit PpIX excretion. Several inhibitors whose modes of action are similar to those of MiTMAB, such as octadecyl trimethyl ammonium bromide (OcTMAB) and RTIL13^48,49^, also inhibited PpIX excretion, while other inhibitors with different modes of action, such as dynole 34–2^48,50^ and Iminodyn 22^48,51^, had little effect on PpIX distribution (data not shown).

### Effects of the double inhibition of ABCG2 and dynamin on PpIX accumulation in JFCR39 cells

**Figure 5:**
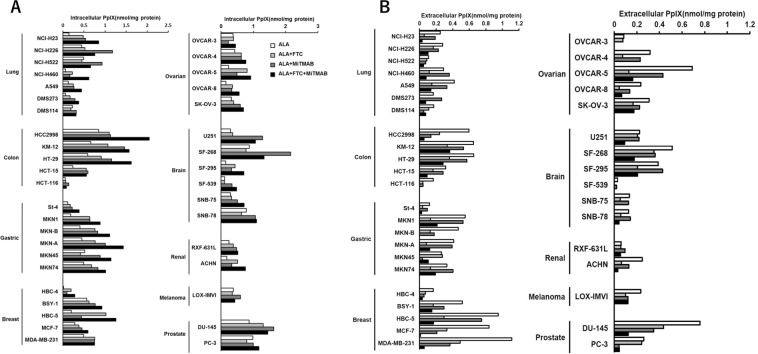
Effects of the double inhibition of ABCG2 and dynamin on PpIX accumulation in the JFCR39 cell lines. (**A**) Intracellular PpIX accumulation in the JFCR39 cell lines after incubation with 1 mM 5-ALA in the presence of FTC and MiTMAB. (**B**) Extracellular PpIX accumulation after incubation with 1 mM 5-ALA in the presence of FTC and MiTMAB. Data are expressed as the means ± S.D. of multiple experimental replicates (n = 3). Dunnett test: *P < 0.05; **P < 0.01.

To investigate the relationship between the ABCG2-dependent pathway and the dynamin-dependent pathway, we next performed a comprehensive study of the combined inhibition of ABCG2 and dynamin with FTC and MiTMAB in the JFCR39 cell panel. Intracellular PpIX levels were increased in almost all cell lines by MiTMAB treatment. The degree of intracellular PpIX increase induced by MiTMAB treatment tended to be greater than that induced by FTC treatment. Moreover, the combined use of MiTMAB and FTC had a much stronger effect on the intracellular accumulation of PpIX compared with that induced by the single inhibitors (Fig. 5A). The effects of the inhibitors on the extracellular PpIX contents in all cell lines were further examined. MiTMAB treatment dramatically decreased extracellular PpIX levels in several of the cell lines tested, and the combination of MiTMAB and FTC efficiently blocked PpIX excretion (Fig. 5B). These results indicated that MiTMAB and FTC inhibited PpIX excretion by different modes of action and that both the ABC transporter pathway and the exocytosis pathway play independent roles in PpIX transportation across the cell membrane.

## Discussion

In this study, we evaluated cancer detection via 5-ALA-derived PpIX accumulation using cancer cells originating from various tissues under the same conditions. We employed the JFCR39 cell panel, which is a well-characterized panel of human cancer cell lines, as a model system. The accumulation of PpIX was confirmed in all the tested cell lines from the JFCR39 cell panel regardless of their tissue origin. These findings strongly suggest that the detection of cancer via 5-ALA-derived PpIX accumulation, which could be applied to PDD and PDT, is feasible at least for all types of cancer that constitute the JFCR39 cell panel: lung cancer, colon cancer, gastric cancer, breast cancer, ovarian cancer, brain cancer, renal cancer, melanoma, and prostate cancer. There have been several *in vitro* studies that have showed the feasibility of using 5-ALA-derived PpIX accumulation for cancer imaging; however, these studies used cells from a limited number of tissues, and the conditions used for cell examination were not identical. Therefore, there were difficulties in determining the feasibility of using 5-ALA-derived PpIX accumulation for cancer imaging for all types of cancer. Here, we were able to confirm the results of those previous studies, and we could also compare the feasibility of using 5-ALA-derived PpIX accumulation for cancer imaging in various cancer cell lines that originated from different tissues. This is the first report that has demonstrated the potential of using 5-ALA-derived PpIX accumulation for cancer imaging *in vitro* in a variety of cancer cells under identical conditions in the presence of 5% FBS.

ALA-PDD has already been approved for concomitant use in the surgical resection of brain glioma^52^ and noninvasive bladder cancer^53^. It has been reported that cell lines established from most types of cancer in which the feasibility of ALA-PDD was shown *in vivo* also accumulated PpIX *in vitro*. Therefore, we anticipate that ALA-PDD is effective in a wider variety of cancers than we tested here, and we should be able to expand its clinical use beyond the scope of the present application.

It has been suggested that the accumulation of PpIX in cancer cells is affected by multiple biological factors, including transporters that mediate the uptake of 5-ALA into cells and excrete PpIX from cells, enzymes in the heme biosynthesis pathway (especially ferrochelatase (FECH)), and the intracellular Fe^2+^ content. PpIX is synthesized in mitochondria from 5-ALA by heme synthesis enzymes. There are no other biochemical pathways that generate PpIX in cells. However, there may be several other pathways involved in eliminating PpIX from cells. For example, it is known that ABC transporters, such as ABCG2, secrete PpIX from cells. Hagiya *et al*. clearly demonstrated that the balance between 5-ALA uptake and PpIX excretion was a substantial factor that determines the intracellular accumulation of PpIX^39^. As shown in Fig. 2A, we detected PpIX both inside and outside of almost all cell lines in the JFCR39 cell panel. These facts suggest that all cancer cells can take up 5-ALA and metabolize 5-ALA to generate PpIX. Thus, in this study, we focused on the mechanisms involved in PpIX excretion.

To date, ABCG2, which is a drug transporter involved in resistance, has been regarded as a cell membrane molecule that plays a major role in excreting PpIX. The role of ABCG2 in the mitochondrial membrane was also discussed by Kobuchi *et al*.^33^. The amounts of ABCG2 determined by Western blot showed that ABCG2 was present in both the plasma and the mitochondrial membranes. Membrane ABCG2 molecules play a role in the excretion of PpIX both from the mitochondria to the cytoplasm and from the cytoplasm to the extracellular lumen. Thus, we comprehensively verified the effect of an ABCG2 inhibitor, FTC, on PpIX accumulation by using the JFCR39 cell panel. In most of the cancer cells in the JFCR39 cell panel, FTC increased the intracellular amount of PpIX and simultaneously decreased the amount of extracellular PpIX (Fig. 2). These results are consistent with the previous observation reported by Zhou *et al*. that intracellular PpIX content and ABCG2 expression are inversely correlated in mature blood cells^54^. Hagiya *et al*. reported a strong correlation between intracellular PpIX accumulation and ABCG2 protein expression in gastric and bladder cancer cell lines^39,55^. These reports strongly suggest that ABCG2 plays an important role in PpIX excretion.

However, the present study provided evidence that extracellular PpIX was still detectable in the majority of the JFCR39 cell lines when ABCG2 activity was suppressed by FTC treatment. We noted that FTC could not completely block the cellular excretion of PpIX in several cell lines in the JFCR39 cell panel. Moreover, we noticed that there were a few cell lines that exported a high level of PpIX despite poor ABCG2 protein expression (Fig. 3B). These findings suggest that there are other pathways besides the ABCG2 pathway that are involved in the excretion of PpIX in cancer cells. Thus, we have conducted extensive studies to determine which transporters are mainly involved in PpIX excretion.

Hagiya *et al*. reported that ABCG2 plays the most important role in PpIX transport of all ATP-dependent transporters^39^. It is unlikely that ATP-dependent transporters other than ABCG2 play such a significant role in PpIX excretion based on their substrate specificities, even though the ABC transporter system is redundant. Considering these facts, we focused on exocytosis, which is another major pathway involved in substance transport across the cellular membrane. To investigate whether exocytosis is involved in PpIX excretion, we attempted to study the effect of exocytosis inhibitors on PpIX excretion. Of the molecules involved in exocytosis, we selected dynamin 2 protein as an inhibition target because dynamin 2 is the master regulator of both cellular membrane endocytosis and exocytosis, and several inhibitors of dynamin 2 with different modes of action are available.

Dynamin was first described as a GTPase that mediates various membrane processes. It is well known that dynamin plays a very important role in clathrin-mediated endocytosis via its ability to separate small vesicles from the cellular membrane. The binding and hydrolysis of GTP is essential for this process^56^. Recently, it was reported that dynamin regulates the size of small pores and is generated by the fusion of a secretion vesicle and the cellular membrane, suggesting that dynamin also plays an important role in the regulation of exocytosis^57–60^. Jackson *et al*. observed that dynamin activators increased secretion via exocytosis from chromaffin cells^61^. Therefore, it is likely that dynamin regulates not only endocytosis but also exocytosis.

A correlation between the dynamin 2 protein expression level and the ability of cells to excrete PpIX was revealed in the present study. Therefore, we expected that exocytosis might be another major pathway involved in the excretion of PpIX from cancer cells. To confirm this hypothesis, we added 5-ALA and a dynamin inhibitor to JFCR39 cell lines and measured both the intracellular and extracellular PpIX contents. The combined treatment of cells with MiTMAB and 5-ALA increased intracellular PpIX levels when compared to treatment with 5-ALA only. MiTMAB could increase intracellular PpIX levels even in the cells with low ABCG2 expression and low sensitivity to FTC. In some cell lines with high sensitivity to FTC, MiTMAB treatment had a stronger effect than FTC treatment. Combined treatment with MiTMAB and FTC increased intracellular PpIX levels much more than treatment with either MiTMAB or FTC alone. We confirmed that the concentrations of inhibitors used were not at cytotoxic levels. Except for 6 cell lines among the 39 cell lines, the intracellular and extracellular levels of PpIX were highest and lowest, respectively, in the case of treatment with both MiTMAB and FTC. In those 6 cell lines, the differences in the intracellular and extracellular PpIX levels in MiTMAB- or FTC-treated only cells and MiTMAB/FTC-treated cells were not significant.

**Figure 6:**
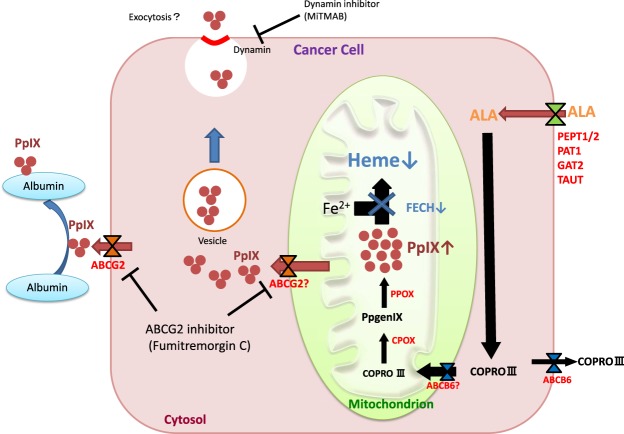
Schematic illustration of PpIX excretion in human cancer cells after the administration of 5-ALA.

These results suggest that the cellular efflux of PpIX involves not only ABCG2 but also exocytosis. Since the inhibition of extracellular efflux by inhibitors of both ABCG2 and exocytosis was not complete in our experiment, other mechanisms must be involved, such as passive diffusion and active transport by unidentified transporters other than ABCG2. Based on our data, we propose that the mechanisms of the excretion of PpIX are as shown in Fig. 6.

The dynamin 2 protein has a structure composed of 5 domains, including (in order from the N-terminus to the C-terminus) the GTP hydrolysis domain, the dynamin middle domain, the Pleckstrin homology domain (PH domain), the GTPase effector domain (GED), and the proline rich domain (PRD),. These domain structures have been investigated and characterized in terms of their roles in dynamin function^62^. All dynamin inhibitors that suppressed PpIX excretion are targeted to the PH domain. Inhibitors targeting the GTP hydrolysis domain, such as Dynole 34–2 and Iminodyn 22, showed no effect on intracellular PpIX levels in our assays.

Based on these results, we anticipate that the PH domain of dynamin 2 is likely to have an essential function in PpIX excretion. The PH domain binds to PI(4,5)P2 in the cellular membrane and controls its distribution. The PH domain has also been reported to regulate dynamin function by enhancing GTPase activity through its interaction with the cell membrane^63,64^.

Since the present study strongly suggested the involvement of dynamin 2 protein in PpIX efflux, we have preliminarily examined the cellular exocytosis of PpIX in human prostate cancer PC-3 and DU-145 cells by using two-photon excitation imaging and observed the exocytosis of PpIX initiated by Ca^2+^ release from the caged-Ca^2+^ compound o-nitrophenyl-EGTA (NP-EGTA, Molecular Probes) upon stimulation with a flash of UV light for 1/16 sec (data not shown)^65^. Red fluorescence from intracellular PpIX decreased within 0.26 sec after the UV flash. In the absence of preincubation with NP-EGTA, such phenomena were not observed upon the UV flash. Further investigation will reveal the precise mechanism by which PpIX is transported via exocytosis that involves dynamin 2.

The efficiency of PDD or PDT depends on the difference in PpIX accumulated in tumor cells and in surrounding normal cells in the relevant target tissue. Therefore, it is important to enhance PpIX accumulation in the targeted tumor cells but not in the surrounding normal cells by inhibiting PpIX excretion. Although the use of *in vitro* studies using cultured tumor cells to speculate on the efficiency of ALA-PDD or ALA-PDT *in vivo* is limited, inhibitors of both the ABCG2 transporter and dynamin-mediated exocytosis could be useful for the enhancement of ALA-PDD or ALA-PDT, and further investigation of those inhibitors for clinical application is needed.

In the present study, we revealed that all human cancer cell lines in the JFCR39 cell panel accumulated PpIX during 5-ALA treatment regardless of their tissue origin. These findings strongly suggest that 5-ALA-derived PpIX accumulation, in principle, can be used for the diagnosis of most types of cancer. It is also known that PpIX that accumulates in cancer cells generates singlet oxygen by excitation with red light. This property has been used for ALA-PDT for diseases that include actinic keratosis and basal cell carcinoma in the skin. Therefore, higher levels of 5-ALA-derived PpIX accumulation in tumor cells compared to those in surrounding normal tissues would be useful for both the diagnosis and therapy of many types of cancer. We observed the possibility that not only ABC transporters but also exocytosis machinery are involved in PpIX excretion from cancer cells. Based on our findings, we expect the development of a better method to increase the concentration of intracellular PpIX and enhance the accuracy of diagnosis and the efficacy of treatment when using ALA-PDD and ALA-PDT, respectively.
